# Patient-derived tumor organoids: advances, applications, and future directions in biomedical research

**DOI:** 10.3389/fmed.2025.1733668

**Published:** 2026-01-14

**Authors:** Ahmet Acar

**Affiliations:** Department of Biological Sciences, Middle East Technical University, Ankara, Türkiye

**Keywords:** 3D culture, biomedical research, organoids, patient-derived tumor organoids, translational research

## Abstract

Patient-derived tumor organoids (PDTOs) have become a key tool in cancer and translational oncology because they are physiologically relevant, 3D *in vitro* systems that preserve the genetic, epigenetic and phenotypic features of patient tumors. PDTOs generated from primary, metastatic surgical resection or biopsy material fill the gap between 2D cultures and animal models. PDTOs have been shown to be more accurate for mimicking disease and treatment response. This review outlines the principles and protocols for PDTO production, characterization and validation with a focus on standardization and reproducibility. PDTOs have been widely applied in oncology and increasingly applied into translational pipelines to model tumor biology, predict therapeutic response, and guide precision medicine strategies. They have shown to be predictive for drug response and are being used as personalized therapeutic avatars. However, several challenges remain, including the limited representation of tumor microenvironment, inter-laboratory variability in protocol adaptation and ethical concerns related to biobanking and data governance. New technologies such as immunological and stromal co-culture systems, organoid-on-chip technologies and multi-omic integration will enhance the use of PDTOs in biomedical research.

## 1 Introduction

Over the past two decades, major advances in tissue engineering and stem cell biology have enabled the development of *ex vivo* models that accurately recapitulate human physiology and diseases. For example, organoid technology has been established from self-organizing, three-dimensional (3D) structures of stem cells and primary tissue (1). Organoids have become a cornerstone of a translational research with their ability to mimic structural, cellular and functional properties of the tissue where they are generated (1). A subgroup of organoids namely Patient-Derived Organoids (PDTOs) have taken a considerable attention with their unique features. PDTOs are established directly from diseased patient tissues and successfully preserve the genetic, epigenetic and phenotypic characteristics of their patient tissue of origin (2). Hence, PDTOs are considered as promising tools to recapitulate diseases, test treatments, and more generally for application in precision medicine (3). In this review, the term PDTOs is used to specifically refer to 3D organoid cultures established directly from primary or metastatic human tumor tissues.

The existing limitations of traditional 2D cell line-based model systems including the lack of cellular differentiation, induced genetic drift, lack of tumor heterogeneity have led to development of cellular model systems capturing healthy or diseased physiologies (4). Despite their historical importance, animal models, interspecies including patient-derived xenografts (PDXs), are increasingly constrained by interspecies biological differences and ethical considerations, which limit their translational predictive power for human diseases (5). For these reasons, PDTOs play a critical role offering a patient-derived, -specific, and scalable approach without big ethical and logistic bottlenecks (2). Recent advances in extracellular scaffold engineering and optimized growth factor combinations have significantly expanded the range of patient tissues suitable to successful organoid generation (6). Similarly, technological advances in next-generation sequencing have enabled the rapid incorporation of PDTOs into genomics frameworks including multi-omics data generation of PDTOs from targeted gene panels, whole-exome sequencing, transcriptomics, epigenomics, proteomics and metabolomics (7). This allowed the discovery and characterization of novel disease mechanisms and therapeutic targets as well as predicting treatment response in PDTOs (8). Furthermore, PDTOs have been a critical instrument in clinical practice as they provided a testing platform for different treatment modalities (9). Accordingly, PDTOs can be considered as functional *in vitro* representatives of individual patients and can be utilized to assess both single-agent and combination drug strategies (10). These characteristics of PDTO technology have changed the role of PDTOs from simple research tool into rapidly growing platform for personalized oncology. This review will summarize the recent literature and advancements in PDTO technology, as well their characterization, utilization and future directions. It should be noted that the review specifically focused on PDTOs that are established directly from patient cancer specimens for applications in oncology, drug screening, and precision medicine. Although organoids derived from induced pluripotent stem cells (iPSCs) represent a highly important platform for studying human development and congenital disorders, a comprehensive discussion of iPSC-derived organoids is outside the defined scope of the present review and therefore not been included.

## 2 Methodology of PDTO generation

**FIGURE 1:**
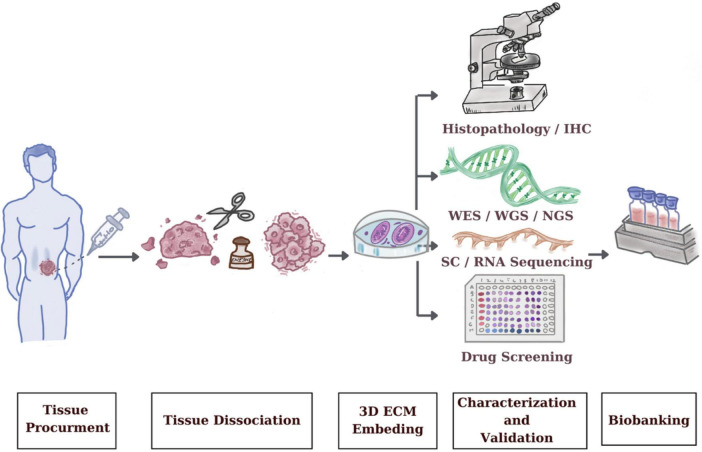
Standard procedure for patient-derived tumor organoids (PDTO) establishment, including tissue acquisition, dissociation, embedding, growing, and characterization.

Methodologies for the establishment of PDTOs are complex since there are number of steps are involved (Figure 1). The critical step for successful PDTO generation begins with the handling and storing the fresh patient specimen in appropriate conditions including preserved cold temperatures and starting PDTO generation within 2–4 h (11). Next, the tissue preparation step begins whereby dissociating the fresh tissue specimen into individual cells (12). After PDTO generation, PDTOs are expanded and at least one subsequent expansion, genetic and phenotypic stability in comparison to initial patient material used for PDTO are confirmed. Molecular, functional and histological characteristics of PDTOs, representative of the tissue origin should be maintained (13). The steps for efficient PDTO generation are linked together, and optimizing each step is crucial for the reproducibility and scalability of the PDTO which maximizes their potential as a platform for personalized oncology.

### 2.1 Tissue procurement and handling

Fresh patient specimens obtained from surgical resections or biopsies constitute the primary source of material for the successful establishment of PDTOs (3). When the surgery is not preferred, core needle biopsies can be used for the establishment PDTOs despite providing a smaller amount of cellular material (9). Regarding the gastrointestinal (GI) PDTOs, performing endoscopic biopsies can lead to derivation of epithelial cells from the stomach, colon or the other regions of the GI track which can then be used for PDTO generation (14). In addition, liquid biopsies, relying on capturing circulating tumor cells (CTCs), have recently become a topic of interest as a minimally invasive approach for the generation of PDTOs (15). The efficiency of PDTO establishment and long-term propagation is strongly influenced by the method of specimen collection, tissue type, and the time elapsed between resection and processing. To maximize this efficiency, tissue processing is recommended to take place within the first 2–4-h window of post-specimen collection accompanied with cold, oxygenated medium specific for PDTOs.

### 2.2 Tissue dissociation

To generate a viable single-cell suspension suitable for PDTO establishment, tumor tissues must undergo carefully optimized mechanical and/or enzymatic dissociation (11). Mechanical tissue separation involves the use of scalpels and scissors to separate the cellular parts (16). Enzymatic tissue separation, on the other hand, utilizes collagenase, dispase, trypsin, or DNase to facilitate the disruption of certain extracellular matrix components and therefore releasing individual cells (16). The duration of enzymatic exposure is a critical parameter, as excessive digestion compromises cell viability whereas insufficient digestion reduces PDTO establishment efficiency (17). Depending on the tissue of origin, the tissue dissociation can vary. For example, very fibrotic stroma present in pancreatic tumor tissue requires prolonged enzymatic exposure time while colon tissue with a more diffuse connective tissue structure requires less enzymatic exposure time for more effective digestion (18). Thus, there is no “one-size-fits-all” approach as each tissue type requires a specific optimization to determine the quality of tissue dissociation for the efficiency of PDTO establishment.

### 2.3 3 D Matrix embedding

Following tissue dissociation, isolated tumor cells are embedded into an extracellular matrix (ECM) that provides both structural support and essential biochemical signals for PDTO self-organization (3). The Matrigel is preferably used ECM derived from Engelbreth–Holm–Swarm mouse sarcoma which is rich of laminin, collagen IV and entactin providing required support and growth for PDTOs (19). This ECM facilitates cell adhesion, proliferation and enables self-organizing for PDTOs (20). Since Matrigel is derived from a murine sarcoma source, it exhibits a batch-to-batch variability, which can significantly affect PDTO morphology, growth rates, and drug response profiles. This variability, in fact, led to the development of synthetic hydrogels as alternatives, offering an opportunity to control mechanical properties and biochemical composition (21). For example, the synthetic hydrogels can be engineered to fine-tune ECM stiffness and present ligands, enabling scientists to study mechanobiology of PDTOs (22). Furthermore, a combination of synthetic hydrogels and natural ECM proteins have emerged, namely hybrid systems, wherein bioactivity and reproducibility can be achieved (23). Therefore, the role of ECM in PDTO generation and maintenance exceeds its role more than as a scaffold and it critically determines the phenotype, lineage specificity and drug response profiles of PDTOs (24). Consequently, a careful selection of ECMs becomes a critical factor for ensuring efficient PDTO establishment and recapitulating patient tissue dynamics.

### 2.4 Defined culture media

Defined organoid culture conditions are formulated to mimic the biochemical signaling mechanisms of the tissue niche and sustain tissue-specific stem cell populations (13). Canonical signaling pathways, including Wnt/β-catenin, epidermal growth factor (EGF), and transforming growth factor-beta (TGF-β), are modulated by defined media supplements to regulate stem cell self-renewal, lineage specificity, and cellular differentiation (13). For example, EGF, R-spondin-1, Noggin, Wnt3a, FGF2, FGF10, A83-01 (a TGF-β inhibitor), and nicotinamide contribute epithelial development and preserve structural organization in PDTOs (13). Tissue-specific requirements for media formulations are taken into consideration for each tissue. For example, colorectal cancer-derived PDTOs rely on Wnt3a and R-spondin to maintain their stem cell compartment (9), gastric PDTOs require gastrin for gastric lineage preservation (14), and prostate PDTOs need androgen to maintain luminal differentiation and secretory function (25). Although media formulations have been so far very well established, the issue of batch-to-batch variation in media formulations containing recombinant proteins still remains as a challenge for the reproducibility of PDTO generation across different research laboratories (26).

### 2.5 Passaging, expansion, and long-term culture

Patient-derived tumor organoids are typically passaged every 7–21 days, depending on their tissue origin, growth kinetics and differentiation status. PDTOs are passaged via mechanical, including pipette-based agitation, and enzymatic digestion which is then followed by re-embedding into an ECM environment supplemented with fresh growth medium. Effective passaging is crucial to overcome overgrowth and media overconsumption related issues and exhibiting long-term stability in culture to preserve genomic and phenotypic characteristics (1). Nonetheless, PDTOs established from normal tissue may encounter problems associated with cellular senescence and thereby exhibiting short-term culture durations (27). Moreover, in some cases, PDTOs do not maintain their phenotypic stability wherein phenotypic shifts occurs (28). Such phenotypic drift is frequently driven by progressive epigenetic reprogramming induced by prolonged *in vitro* culture conditions (29). Therefore, it is critical to validate ongoing culture effects in PDTOs to effectively preserve the original tissue representation.

### 2.6 characterization and validation

Unlike pluripotent stem cell-derived organoid systems, where karyotype anaylysis is a primary quality control step, validation of PDTOs is primarily based on histopathological, genomic, transcriptomic, and functional concordance with the parental tumor tissue (3). It is essential to demonstrate that PDTOs accurately recapitulate the molecular, histopathological and functional characteristics of their parental tumors. To verify that PDTOs resembles the donor tissue, a comprehensive analysis including morphological, molecular and functional validations are performed (3). For example, the glandular architecture and polarity of PDTOs are compared and validated across the donor tissue sections using histopathology analysis (30). Immunohistochemistry is used to confirm lineage-specific biomarkers including CK20 and CDX2 in colorectal cancer PDTOs (9). To validate the genomic resemblance between PDTOs and the donor tissue, whole-genome, whole-exome, targeted panel sequencing or long read sequencing can be employed (31, 32). This approach is based on the assessment of the stability of somatic mutations and mutational burden (9). Another critical molecular validation is that transcriptional profiling through RNA sequencing to verify stable gene expression and signaling activities across the PDTO and the donor tissue (33). Lastly, functional assays assess whether PDTOs exhibits drug sensitivity for oncological PDTO applications (18, 34, 35). Collectively, these validation methods provide a comprehensive approach for assessing the reliability of a PDTO model, and thereby offering a valuable resource for both basic research and translational applications in precision medicine.

### 2.7 Biobanking and sharing

Biobanking efforts for PDTOs have emerged as critical infrastructure for functional precision oncology by enabling systematic generation, storage, molecular annotation, and large-scale drug testing of patient-specific tumor models. In precision medicine workflow, PDTO biobanks are not only repositories for long-term storage but are increasingly established as functional biobanks that integrate clinical metadata, multi-omics profiling, and high-throughput drug screening pipelines. These efforts also enable researchers to perform independent PDTO culturing, validating the reproductivity of culture conditions (36). PDTOs in the culture are harvested for their subsequent freezing which includes a suspension in a freezing media containing 10% dimethyl sulfoxide (DMSO) (11). After PDTOs are mixed with the freezing solution, the samples are subjected to controlled rate of decrease in the freezing before long-term storage in the liquid nitrogen tank (11). Efficient cryopreservation of PDTOs enables the preservation of organoids for their functionality in the biobanks when they are shared and used for additional experiments by other researchers. Importantly, in functional PDTO biobanks, thawed organoids are routinely re-expanded for systematic testing of standard-of-care therapies as well as investigational and targeted compounds, enabling real-time assessment of patient-specific drug sensitive. The resulting functional drug response profiles can be integrated with molecular signatures and, in selected clinical settings, used to support therapeutic decision-making. International efforts for PDTO biobanking such as the Human Cancer Models Initiative and the European Organoid Resource offer established standardization in biobanking, facilitating quality control, electronic health records for clinical, histological and genomic data (37). These coordinated platforms provide not only physical access to well-annotated PDTO models but also integrated clinical, histopathological, and genomic datasets, enabling reproducible drug testing across laboratories and accelerating translational research. As a result, modern PDTO biobanks now function as living resources that bridge basic research, drug discovery, and personalized oncology by continuously linking patient material with functional therapeutic testing and clinical outcome data.

## 3 Applications of patient-derived organoids

The applications of PDOs span from oncology to infectious diseases, genetic disorders and regenerative medicine (38). PDTOs preserve the intratumour heterogeneity, molecular and functional features of the donor tissue they are derived, exhibiting their utilization in both basic and translational research (39). In oncology, the utilization of PDTOs helped the understanding of tumor initiation, progression and treatment response, providing expanded insights into cancer biology and the mechanisms of treatment resistance (3). In infectious diseases, for example, respiratory tract-derived PDOs, namely airway epithelium PDOs, have been instrumental to investigate pathogen-host interactions in a controlled environments mimicking a physiologically similar tissue (40). PDOs have shown to be successful tools representing disease phenotypes for genetic disorders wherein functional assays, gene correction procedures, and preclinical testing of therapies were performed (41). Lastly, in regenerative medicine, organoids established from healthy tissue offers a platform to repair or replace organs for tissue engineering and cell therapy-based applications (42). Thus, the wide range of PDTO technology show the benefits of organoids to uncover novel approaches and integrate with other disciplines to boost both research and clinical practice.

### 3.1 Cancer modeling

Patient-derived tumor organoids pre-present a cornerstone of contemporary cancer modeling by preserving tumor architecture, epi(genetic) heterogeneity, and therapy response profiles (43). For example, the functional consequences of recurrent driver mutations, such as *APC*, *KRAS*, *TP53* in colorectal cancer have been systematically investigated using PDTO platforms (44). Moreover, the relationships between oncogenic signaling and tumor growth dynamics have been studied using PDTOs in a genetically or pharmacologically altered conditions (45). Similarly, PDTOs originating from pancreatic ductal adenocarcinoma (PDAC) showed significant potential, reflecting the chemoresistance seen in patients and indicating as a promising predictive model for effective treatment combinations (46). In addition, triple-negative breast cancer PDTOs helped to test number of different customized therapy options (47). Taken together, PDTOs used in modeling of cancer contributes to the functional analysis of tumor biology within a patient-specific framework and serve as a platform for testing treatment modalities.

The tumor microenvironment (TME) plays a critical role in regulating tumor growth, immune evasion, metastasis, and therapeutic resistance (48). While traditional PDTO cultures were established using epithelial tumor cells, selected number of recent research have shown that incorporation of stromal and immune components significantly boosts the physiological relevance of PDTOs (49). Co-culture systems combining cancer-associated fibroblasts (CAFs) with PDTOs have been shown to modulate tumor proliferation, ECM remodeling, and drug sensitivity (50). Similarly, PDTO-immune cell co-culture platforms enable functional interrogation of immune checkpoint blockade, tumor immune presentation, and mechanisms of immune evasion (48). In addition, incorporation of endothelial cells into PDTO cultures enables angiogenic signaling and provides nutrient diffusion and drug delivery observed in *in vivo* (51). Collectively, these advances demonstrate that accurate modeling of the TME is essential for reproducing patient-specific therapeutic responses and resistance mechanisms. The integration of stromal, immune, and vascular components into PDTO systems is therefore increasingly recognized as a prerequisite for effectively translating PDTO-based discoveries into clinical oncology.

### 3.2 Drug screening and precision oncology

Patient-derived tumor organoids have proven to be critical preclinical model for drug screening and precision oncology (38). Hundreds of therapeutic compounds, either as monotherapies or rational combinations, can be systematically evaluated using high-throughput PDTO-based drug screening platforms (52). Since PDTOs preserve all major characteristics of the donor tissue, HTS using PDTOs facilitate more physiologically relevant platform than conventional two-dimensional cell lines and has demonstrated strong predictive accuracy in clinical research. For example, a pioneering study by Vlachogiannis et al. reported that there was over 80% concordance between the drug response in PDTOs and metastatic GI patients, demonstrating their efficacy as a functional indicator for treatment selection (9). When combined with comprehensive genomic profiling, high-throughput drug screening data derived from PDTOs can be integrated with molecular signatures sensitivity or resistance to guide rational therapeutic selection. Moreover, PDTOs can offer adaptive therapy testing, wherein the consequences a sequential drug testing is monitored for the evolution of resistance (53). Collectively, PDTOs are considered as “living patient avatars” potentially helping to connect molecular diagnoses with individualized treatment plans, so contributing to the improvement of therapeutic accuracy (54).

Besides drug screening, PDTOs have been instrumental for the identification and functional validation of predictive biomarkers of drug sensitivity and resistance, which is a key factor for precision oncology. For example, *KRAS* and *NRAS* mutations have been associated with intrinsic resistance to EGFR-targeted therapies such as cetuximab and panitumumab in colorectal cancer PDTOs (9). On the other hand, *BRAF V600E* mutations have been shown to correlate with reduced response to standard chemotherapy but increased sensitivity to combined BRAF-MEK inhibition in such models (44). In addition, *PIK3CA* mutations and *PTEN* loss detected in PDTOs have been linked to an increase in the response to PI3K-AKT-mTOR signaling pathway inhibitors (47). In pancreatic ductal adenocarcinoma PDTOs, *BRCA1*, *BRCA1* and *PALP2* mutations leading to alterations in DNA damage repair machinery have emerged as critical biomarkers of sensitivity to platinum-based chemotherapy and PARP inhibitors (18). Conversely, distinct transcriptional subtypes identified from RNA sequencing of PDTOs have been associated with differential sensitivity to gemcitabine, FOLFIRINOX, and MEK inhibitors (18). In breast cancer PDTOs, *ER*, *PR*, and *HER2* expression levels maintain their predictive power for endocrine therapy and HER-directed treatments (35). Likewise, in prostate cancer PDTOs, androgen receptor (AR) amplification and splice variants, such as AR-V7, have been linked to resistance to androgen signaling inhibitors (25). Taken together, these examples, demonstrate that PDTO models not only recapitulate patient-specific drug responses but also provide a powerful system for the functional validation of genomic, transcriptomic, and phenotypic biomarkers of drug sensitivity and resistance with an ultimate aim to strength their role in biomarker-driven precision oncology.

### 3.3 Infectious disease models

Organoid systems are considered highly disease-relevant models since they reproduce human epithelial biology, including host-pathogen interactions, under controlled experimental conditions (40). In virology, the reproduction of human norovirus has been demonstrated, for the first time, in human intestinal organoids. Specifically, in this study, viral entry, replication and host organoid response was uncovered (55). Furthermore, airway epithelial-derived organoids have been utilized to investigate respiratory diseases, specifically in relation to the role of the ACE2 receptor and proteolytic cleavage mediated by SARS-CoV-2 (56), which has also been documented, in a separate study, as a critical factor in GI cancers (57, 58). Lastly, gastric organoid models have been developed for simulating Helicobacter pylori infection wherein, as a result, epithelial cell damage, cytoskeletal reorganization, and activation of proinflammatory signaling pathways have been studied (59). These studies show the power of organoids in studying complex host–microbe interactions that traditional culture systems or animal models were unable to facilitate.

### 3.4 Regenerative medicine

Patient-derived organoids (PDOs) have been broadly considered as supporting scaffolds for tissue regeneration and functional restoration in regenerative medicine (42). For example, wounded mice were examined after healthy liver PDOs transplantation and as a result, partially restored liver function was observed, demonstrating the promise of organoid-based hepatocyte replacement therapy (60). Furthermore, corneal epithelial-derived PDOs were into animal models with corneal injury and as a consequence, transparency was restored, indicating a promising vision-restoration therapy (61). Moreover, PDOs from intestinal tissue transplanted into an animal model of ulcerative colitis resulted in mucosal healing with restoration of epithelial barriers, indicating the role of PDO-based therapies for chronic inflammatory diseases (62). Despite these promising studies, there are still challenges associated with PDO-based approaches in regenerative medicine including the organoid production rate, immunological compatibility, and integration into host tissues (50).

## 4 PDTOs vs. other preclinical models

**FIGURE 2:**
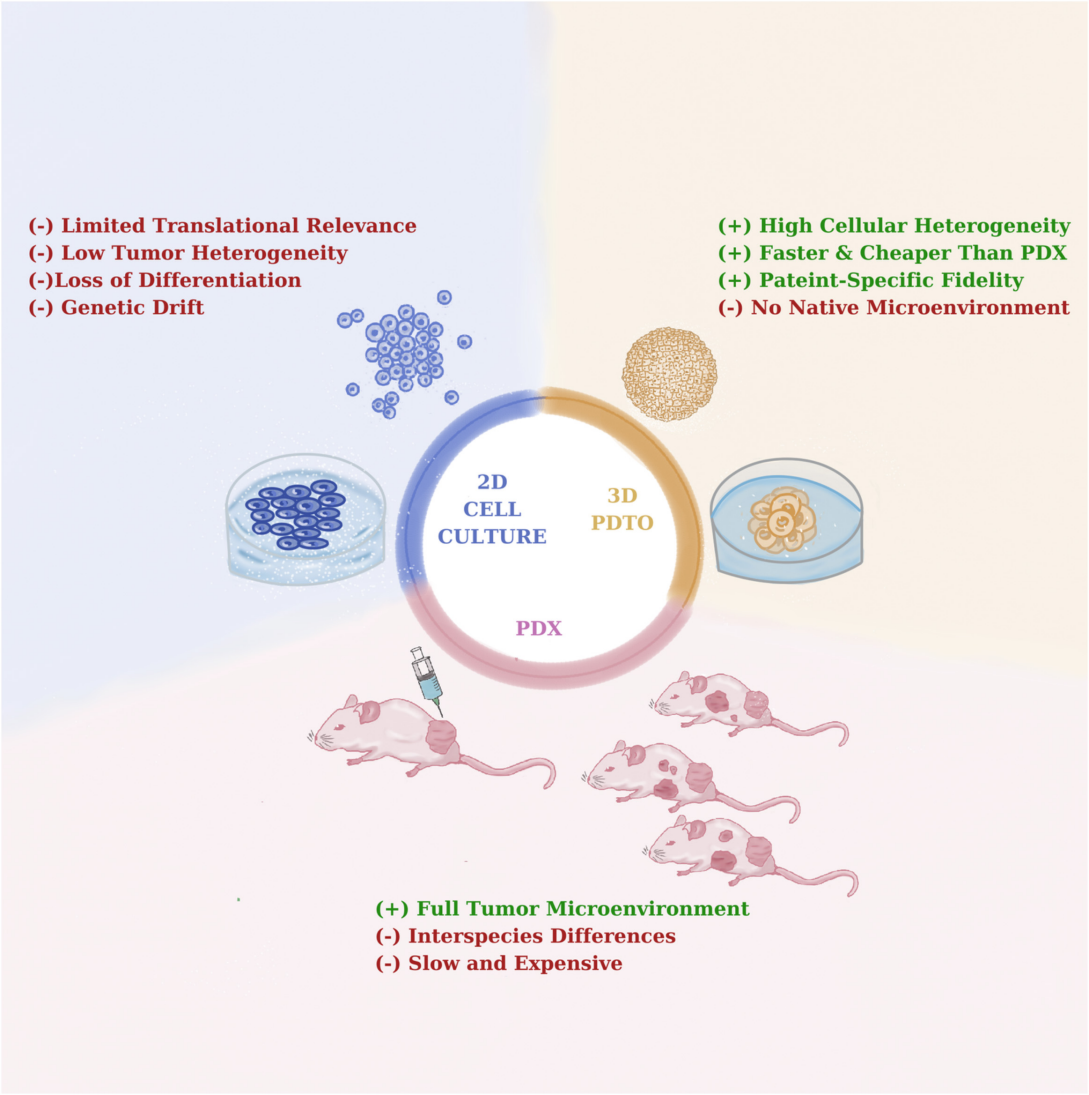
Comparative overview of 2D, patient-derived tumor organoid (PDTO), and patient-derived xenografts (PDX) models.

Among available preclinical cancer models. PDTOs hold a critical intermediate position between traditional two-dimensional (2D) cultures and *in vivo* patient-derived xenografts (PDXs) systems (Figure 2). In comparison to 2D cell line systems, PDTOs demonstrate better cellular differentiation and intratumoural heterogeneity characteristics as well as maintaining structural architecture and lineage specificity of donor tissues (4). Moreover, genetic drift may be prevalent in 2D cell line models resulting in activated signaling pathways and accumulation of genetic artifacts (63). When compared to *in vivo* PDX model systems, PDTOs have been remarkable in terms of offering economic and logistic advantages alongside with scalability for HTS applications (64). PDXs have been reported to provide preserve a tumor microenvironment, including the stroma and vasculature; yet, the presence of murine microenvironment, tumor-host interactions may be impeded, thus affecting the assessment of the treatment response (5). Collectively, it is critical to note that PDTOs and PDXs should be regarded as complimentary systems. For example, PDTOs can provide fast preliminary drug screening to identify promising candidate therapies, which can subsequently be tested using PDXs for their *in vivo* efficacy. (65). This integrative strategy maximizes the efforts in terms of time and resources, while also improving the predive power of such systems in preclinical drug development processes.

## 5 Limitations and current challenges

All advantages and limitations discussed in this section refer specifically to PDTOs and their applications in cancer research. Although PDTOs offer substantial translational potential, several biological, technical, and logistic challenges currently limit their universal clinical implementation. For example, PDTOs lack intact microenvironment including its components such as endothelial cells, cancer-associated fibroblasts, immune cells (66). The lack of these stromal cell types limits the capacity of PDTOs to recapitulate heterotypic cell-cell interactions observed in tumor tissues. Furthermore, the success rates for establishment of PDTOs vary depending on the donor tissue type such as sarcomas exhibiting lower PDTO generation rates due to their distinctive stroma and cellular characteristics (26). Another constrain is due to the lack of protocols for tissue handling, culture media conditions, accepted by other laboratories which may impact the reproducibility of PDTO establishment (50). Reproducibility is a critical constrain for the reliable application of PDTOs in basic research, translational research, and clinical decision making (50). Variability in PDTO establishment and consecutive experimental outcomes can arise from multiple sources including differences in tissue procurement, enzymatic dissociation protocols, ECM composition, and growth factor formulations (11). In particular, batch-to-batch variability of Matrigel and recombinant growth factors represents one of the contributors to inter-laboratory inconsistencies in PDTO growth dynamics, morphology, and drug response profiles (21). Moreover, the high costs associated with culture media supplements, including recombinant growth factors, and ECM, namely the Matrigel, can be considered as additional constrains associated with PDTO establishment. To address these issues, synthetic hydrogels are increasingly adopted as alternative sources to animal-derived matrices to improve the standardization and experimental outputs (22). In parallel, the harmonization of culture conditions through consensus media formulations and the implementation of standardized quality control checkpoints, including genomic instability, histopathological, and functional drug testing which significantly improved the reproducibility and cross-laboratory comparability of PDTO models (29). Furthermore, ethical approval processes linked to obtaining the donor samples can form significant barriers in terms of time and logistics, especially if the ultimate purpose of PDTO establishment is toward the biobanking (36). Lastly, informed consent before sample collection, data protection and the presence of governance guidelines for sharing PDTOs and associated clinical data must be established before initiating PDTO-based research or a clinical study.

## 6 Emerging innovations and future directions

**FIGURE 3:**
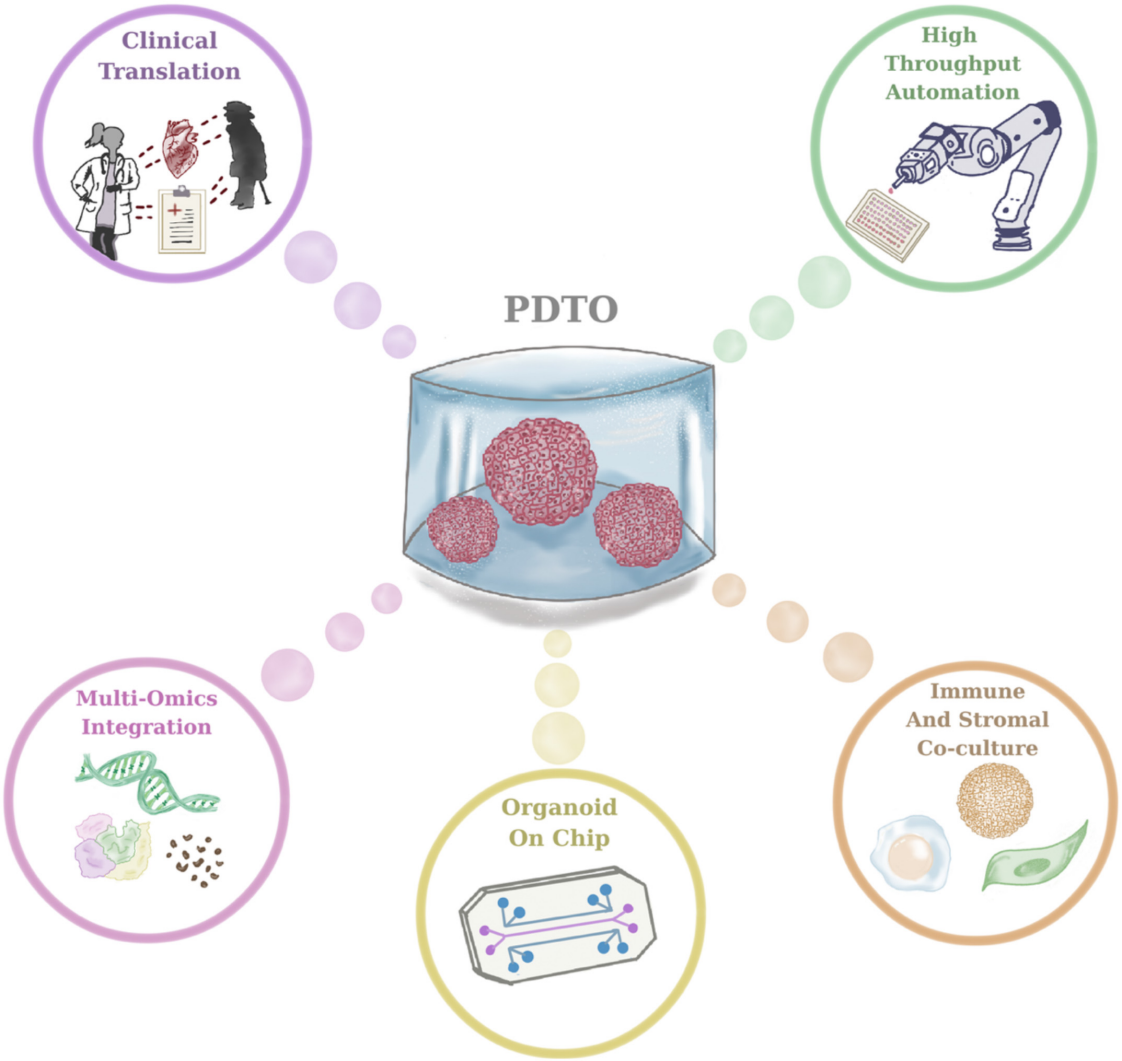
Future directions in patient-derived tumor organoid (PDTO) research and clinical translation.

Emerging technological advancements are expected to further refine PDTO platforms and accelerate their integration into precision oncology workflows (Figure 3). Critical need lies in integrating immune and stromal cells in PDTO cultures, enabling the establishment of co-culture systems which more precisely resemble tumor tissue dynamics (49). For example, co-culture systems based on immune cells and PDTOs have been utilized to study immune checkpoint blockage and immune evasion mechanisms (48). Another rapidly advancing area is organoid-on-chip systems, relying on the integration of PDTOs with microfluidic chips that facilitate the monitoring of dynamic media flow, mechanical stimuli and multi-tissue interfaces (67). Another emerging field in relation to HTS using PDTOs is that robotic platforms allow high-throughput automation to standardize medium exchange, imaging, and handling. Additionally, clinical trials, namely the SENSOR study, is critical for the assessment of the feasibility and subsequent development of PDTO-based therapeutic intervention strategies for clinical workflow. Another emerging direction in 3D cancer modeling is the development of assembloids, which are composite 3D systems generated by the controlled integration of multiple organoid types or the combination of PDTOs with stromal, immune, or neural components (68). In the context of cancer research, tumor assembloids enable the reconstruction of complex tumor-microenvironment interactions, including metastatic niche formation and immune infiltration (69). The integrations of PDTOs into assembloid systems is therefore expected to further enhance the translational relevance of organoid-based cancer modeling. Lastly, cutting-edge approaches integrated with PDTOs, namely cellular barcoding technology, will be instrumental to enhance the understanding about the underlying mechanisms of drug resistance (70–73). Taken together, these advancements offer promising solutions to improve the understanding and contribution of PDTOs into clinical practice with more standardized and personalized approaches for near future.

## 7 Conclusion

In conclusion, PDTOs constitute a transformative platform for biomedical research and precision oncology by accurately preserving patient-specific tumor biology *in vitro*. PDTOs can offer unparallel scalability, experimental adaptability, and biological relevance to human tumors when compared to conventional *in vitro* and *in vivo* model systems. Despite the numerous advantages of PDTOs, they still require improvements such as the absence of microenvironment, inconsistencies and variability in the establishment of PDTO protocols from diverse tissue types. The development of co-culture systems, bioengineered matrices, microfluidics, and high-throughput techniques holds significant promise in mitigating these challenges. The improvements in the culture conditions, enhanced reproducibility capacity across different laboratories, and increased transparency of regulatory bodies for integrating PDTOs into the clinic will facilitate the transition of PDTOs from research laboratories into clinical settings that will guide the clinical decision-making.
